# Digitale Interventionen für Geflüchtete. Herausforderungen, Chancen und die Perspektive der *agency*

**DOI:** 10.1007/s00481-021-00621-6

**Published:** 2021-03-26

**Authors:** Giovanni Rubeis

**Affiliations:** Institut für Geschichte und Ethik der Medizin, Medizinische Fakultät, Ruprecht-Karls-Universität Heidelberg, Im Neuenheimer Feld 327, 69120 Heidelberg, Deutschland

**Keywords:** Agency, Digitale Interventionen, Geflüchtete, mHealth, Mental Health, Migration, Agency, Digital interventions, Mental health, mHealth, Migration, Refugees

## Abstract

Geflüchtete weisen eine hohe Prävalenz an psychischen Störungen auf. Dem hohen Behandlungsbedarf stehen jedoch Barrieren gegenüber, die den Zugang zu psychischen Versorgungsleistungen behindern. Zu den Zugangsbarrieren gehören strukturelle Hürden ebenso wie kulturell differente Haltungen gegenüber psychischer Gesundheit und Krankheit sowie therapeutischen Maßnahmen. Eine Möglichkeit, diese Zugangsbarrieren zu überwinden und Geflüchteten Versorgungsleistungen nach ihrem Bedarf zukommen zu lassen, wird in digitalen Interventionen gesehen. In Form von interaktiven Websites oder Smartphone-Apps haben sich diese internet- und mobilgestützten Interventionen bereits in der Versorgung bewährt. Auch gibt es erste Beispiele für einen gelungenen Einsatz bei Geflüchteten. Die ethischen Aspekte digitaler Interventionen für Geflüchtete sind bislang aber kaum erforscht. Ziel des Beitrags ist es, dieses Desiderat zu bearbeiten. Als Instrument der ethischen Analyse wird dazu das *agency*-Konzept verwendet. Nach dem *agency*-Konzept sind Personen als handlungsfähige Akteure zu verstehen, die aus eigenen Ressourcen und Kompetenzen schöpfen und selbstwirksam handeln können. *Agency* ist in vorliegender Arbeit das Leitprinzip, um die Chancen und Risiken digitaler Interventionen bei Geflüchteten zu analysieren. Darüber hinaus werden die Perspektiven eines *agency*-basierten Einsatzes digitaler Interventionen für Geflüchtete aufgezeigt. Auf dieser Grundlage können therapeutische Konzepte entwickelt werden, die aus ethischer Sicht zu einer Verbesserung der Versorgungssituation von Geflüchteten beitragen können.

## Abstract (en)

**Definition of the problem:**

Refugees show a high prevalence of mental health burden. Catering to the need for mental health services is made difficult by access barriers. These barriers consist of structural factors as well as culturally different attitudes towards mental health, mental illness, and therapeutic interventions. One option to overcome these access barriers and to provide mental healthcare services in an appropriate manner is seen in digital interventions. In the form of interactive websites or smartphone apps, these interventions have proven to be effective in mental healthcare. There are also promising examples of successful use of these technologies with refugees. However, the ethical aspects of digital interventions for refugees have scarcely been studied yet.

**Arguments:**

Aim of this paper is to tackle this research desiderate. The instrument of the ethical analysis to be conducted is the concept of agency. According to the concept of agency, persons have to be seen as actors capable of acting based on their own resources and competencies. Agency is the guiding principle here for analyzing the opportunities and risks of digital interventions for refugees. In addition, I will attempt to point out prospects for an agency-based application of digital interventions.

**Conclusion:**

The ethical analysis could be used as the blueprint for developing therapeutic concepts that contribute to the improvement of mental healthcare services for refugees from an ethical perspective.

## Einleitung

Durch die Covid-19-Pandemie und die damit verbundenen Engpässe in der Gesundheitsversorgung ist auch die Versorgungssituation von Geflüchteten wieder in den Fokus geraten. Gerade hinsichtlich der psychischen Versorgung von Geflüchteten werden Schwierigkeiten und Herausforderungen berichtet, die auch zukünftig bestehen werden. Dazu gehört zunächst die im Vergleich zur Gesamtbevölkerung hohe Prävalenz psychischer Störung in der Population der Geflüchteten (Böttche et al. 2016; Georgiadou et al. 2018; Metzing et al. 2020; Morina et al. 2018; Satinsky et al. 2019). Besondere Häufigkeit weisen dabei Traumafolgestörungen wie die posttraumatische Belastungsstörung (PTBS) (Böttche et al. 2016; Georgiadou et al. 2018; Morina et al. 2018; Rometsch-Ogioun El Sount et al. 2018; Sijbrandij et al. 2017; Sukale et al. 2017; Witt et al. 2015) und affektive Störungen, v. a. Depression auf (Böttche et al. 2016; Georgiadou et al. 2018; Kandiah 2018; Morina et al. 2018; Sukale et al. 2020; Schneider et al. 2017; Sijbrandij et al. 2017). Darüber hinaus gibt es unter den Geflüchteten hochvulnerable Gruppen mit besonderem Behandlungsbedarf. Dazu gehören Frauen, die Opfer von Gewalt wurden (Bajbouj et al. 2018; Rometsch-Ogioun El Sount et al. 2018; Schneider et al. 2017) sowie Minderjährige (Buchmüller et al. 2018; Sijbrandij et al. 2017; Sukale et al. 2017, 2020; Walg et al. 2017; Witt et al. 2015). Neben den Einflussfaktoren vor und während der Flucht sind besonders Postmigrationsstressoren ausschlaggebend für die hohe Prävalenz (Böttche et al. 2016; Burchert et al. 2019; Georgiadou et al. 2018; Kandiah 2018). Dazu gehören strukturelle Faktoren wie unsichere Aufenthaltsperspektive, unklarer Aufenthaltsstatus, langwierige Asylverfahren, fehlende Arbeitserlaubnis, eingeschränkte soziale Teilhabe und eingeschränkter Zugang zur Gesundheitsversorgung, kulturelle Faktoren wie Sprachbarrieren und individuelle Faktoren wie die Trennung von Familienmitgliedern, Diskriminierung und Einsamkeit (Böttche et al. 2016; Kandiah 2018; Rometsch-Ogioun El Sount et al. 2018; Wagner 2016).

Dem hohen Bedarf der Geflüchteten nach psychischer Versorgung stehen Zugangsbarrieren zu Versorgungsangeboten im deutschen Gesundheitswesen gegenüber. Hier sind rechtliche Beschränkungen des Zugangs zu Versorgungsleistungen, Sprachbarrieren und das Fehlen von Dolmetschern sowie Personal mit interkulturellen Kompetenzen zu nennen. Zudem werden vorhandene Versorgungsangebote aufgrund kulturell differenter Haltungen gegenüber psychischen Erkrankungen und psychischer Behandlung sowie der Angst vor Stigmatisierung oftmals nicht wahrgenommen. Somit besteht die doppelte Problematik des mangelnden Angebots psychischer Versorgungsleistungen und der in manchen Fällen mangelnden Bereitschaft, vorhandene Angebote wahrzunehmen.

Um diese zweifach bedingte Versorgungslücke zu überwinden, wird in der Forschungsliteratur häufig der Einsatz von digitalen Interventionen diskutiert (Böttche et al. 2016; Heim und Burchert 2018; Schneider et al. 2017; Martin-Shields 2017; Preitler 2018). Digitale Interventionen erlauben den orts- und zeitungebundenen Zugang zu psychischen Versorgungsleistungen, worin die Verringerung einer zentralen Zugangsbarriere gesehen wird. Hinzu kommt, dass gerade für die Wirksamkeit von digitalen Interventionen in der Behandlung von Depression und PTBS, die häufigsten Störungen bei Geflüchteten, eine umfangreiche Evidenz vorliegt (Callan et al. 2017; Kerst et al. 2019; Sijbrandij et al. 2016). Auch wurden digitale Interventionen bereits in der Population der Geflüchteten eingesetzt und damit positive Resultate erzielt (Ashfaq et al. 2020; Burchert et al. 2019; Heim und Burchert 2018; Sijbrandij et al. 2017). Demnach haben sich digitale Interventionen als Möglichkeit erwiesen, einen niedrigschwelligen Zugang zu psychischen Versorgungsleistungen zu ermöglichen, und könnten dadurch die Versorgungssituation insgesamt verbessern.

Die ethischen Aspekte des Einsatzes digitaler Interventionen für Geflüchtete sind bislang allerdings kaum erforscht. Zwar werden bestimmte ethische Aspekte, beispielsweise der Aspekt der Kultursensibilität von Anwendungen, gelegentlich in empirischen Untersuchungen diskutiert. Eine systematische ethische Analyse steht jedoch noch aus. Der vorliegende Beitrag ist als Versuch zu verstehen, dieses Desiderat zu bearbeiten.

Als handlungsleitendes ethisches Prinzip wird dabei der *agency*-Ansatz vorgeschlagen. *Agency* ist ein zentraler Begriff der sozialwissenschaftlichen (Giddens 1984; Emirbayer und Mische 1998; Sewell 1992) sowie der philosophischen Handlungstheorie (Sen 1992, 1999). Mittlerweile kann man auch von einem Paradigma sprechen, also einem Leitbild, das eine fruchtbare Heuristik menschlichen Handelns bietet (Mick 2012). *Agency* beschreibt das Vermögen von handelnden Personen (Akteuren), durch ihre individuellen Kompetenzen, Ressourcen und Wertvorstellungen die Strukturen ihrer Umwelt zu reproduzieren und zu modifizieren. Dabei können Akteure durch Verfolgen eigener Ziele und Wertvorstellungen Probleme bewältigen und strukturelle Schwierigkeiten überwinden. Wichtig ist hierbei, Personen als handlungsfähige Akteure zu begreifen, die aus eigenen Ressourcen und Kompetenzen schöpfen und selbstbestimmt handeln können. Auch in psychischen Versorgungsansätzen spielt *agency*, verstanden als Selbstwirksamkeit, eine Rolle. Prominentes Beispiel ist die klientenzentrierte Psychotherapie nach Rogers (Rogers 1951, 1961).

Für die Verwendung des *agency*-Konzeptes im Rahmen der ethischen Analyse von digitalen Interventionen bei Geflüchteten sprechen v. a. drei Gründe. Erstens werden Geflüchtete oftmals als Opfer (von Folter, Vertreibung, Gewalt) angesehen und damit in die passive Rolle von Hilfsbedürftigen gedrängt (Geiger 2016; Kandiah 2018; Preitler 2018). Das *agency*-Konzept bricht diese passive Opferrolle auf und zielt darauf, vorhandene Ressourcen und Kompetenzen aktiv zu nutzen (Geiger 2016). Damit wird auf die Handlungsfähigkeit der involvierten Akteure aufgebaut. Somit lässt sich auf der Grundlage von agency für eine *ressourcenorientierte Versorgung* argumentieren.

Zweitens bietet das *agency*-Konzept im Kontext psychischer Störungen einen Vorteil gegenüber dem Prinzip der Autonomie, das zumeist im Fokus medizinethischer Analysen steht. Im psychotherapeutischen Kontext versteht man unter Autonomie zumeist Selbstbestimmung und Selbstregulation, wobei der Aspekt eigenverantwortlichen Handelns betont wird (Lindenmeyer 1999). Bei psychischen Erkrankungen kann allerdings die Fähigkeit zur Autonomie durch das Störungsbild bzw. die Symptomschwere beeinträchtigt sein (Hinterhuber 2017). Oftmals ist die (Wieder‑)Herstellung dieser Fähigkeit ein Therapieziel. Autonomie kann daher oftmals nicht global zu- oder abgesprochen werden, sondern ist situationsbezogen zu beurteilen (Scholten und Vollmann 2017). Hinsichtlich digitaler Interventionen ergibt sich oftmals ein Spannungsfeld zwischen der Autonomie und dem Wohl von Klient*innen (Rubeis und Ketteler 2020). Da digitale Interventionen zumeist auf Selbstmanagement zielen, müssen sie auf die individuellen Bedarfe und Ressourcen von Klient*innen abgestimmt sein. Eine übersteigerte Selbstwirksamkeitserwartung kann bei ausbleibendem Therapieerfolg zu Frustrationserfahrungen und Symptomverschlechterung führen (Westermann et al. 2017). Somit ist es sinnvoller, das weniger voraussetzungsreiche Konzept der *agency* als ethisches Analyseinstrument zu verwenden. Agency zielt zwar auch auf Selbstwirksamkeit, bezieht sich dabei aber auf die jeweils vorhandenen individuellen Bedarfe. Somit lässt sich das *agency*-Konzept flexibler anwenden als das Konzept der Autonomie und kann als Grundlage für eine *bedarfsorientierte Versorgung* herangezogen werden.

Drittens ist das *agency*-Konzept auch in kultureller Hinsicht weniger voraussetzungsreich als das Konzept der Autonomie. Im Vergleich zum westlich-individualistischen Verständnis der Autonomie, das nicht in allen Kulturen akzeptiert ist, kann das *agency*-Konzept auch in einem stärker familiär- bzw. gemeinschaftlich ausgerichteten Setting verwirklicht werden. Hierfür bietet sich ein Konzept von *agency* an, das sich als intrinsisch sozial und relational versteht (Emirbayer und Mische 1998). Anstelle eines Autonomie-Konzepts, das dem Individuum feststehende Eigenschaften zuschreibt, versteht dieser Ansatz das handelnde Individuum maßgeblich als durch Strukturen, Prozesse und soziale Relationen bedingt (Geiger 2016). *Agency* lässt sich somit als flexibles Prinzip auf kulturell diverse Handlungskontexte anwenden, ohne substantialistische Vorannahmen jenseits der Handlungsfähigkeit des Subjekts als Minimalbedingung treffen zu müssen. Das Konzept der *agency* lässt sich durch diese Flexibilität vor dem Hintergrund anwenden, dass Geflüchtete eine in sich heterogene Gruppe mit unterschiedlichen soziokulturellen Beziehungsformen darstellen. Im Hinblick auf eine *kultursensible Versorgung* kann das *agency*-Konzept daher einen fruchtbaren Ansatz darstellen.

Im Folgenden werden in einem ersten Schritt die Chancen digitaler Interventionen für Geflüchtete analysiert. In einem weiteren Schritt werden die Risiken diskutiert. Leitbild ist hierbei jeweils das *agency*-Konzept. Nach der Analyse der Chancen und Risiken wird die Einbettung von digitalen Interventionen in ein *Stepped-and-Collaborative-care-*Modell (SCCM) als Perspektive diskutiert, wobei *agency* das ethische Paradigma darstellt. Auf dessen Grundlage werden die Perspektiven einer kultursensiblen, bedarfsorientierten und ressourcenorientierten Versorgung erarbeitet. Dem Vorgehen liegt ein konsequentialistischer Ansatz zugrunde, wonach Nutzen und Risiken einer Handlungsalternative (digitale Intervention) zu einer bestehenden Praxis (derzeitige Versorgungsformen) abgewogen werden.

In dieser Arbeit wird der Begriff „Geflüchtete“ für Personen verwendet, die nach ihrer Ankunft in Deutschland ein Asylgesuch gestellt haben. Der Begriff „Geflüchtete“ umfasst hier auch Asylsuchende und Geduldete, mithin Personen ohne anerkannten Schutzstatus. Damit folge ich der Begrifflichkeit, wie sie im aktuellen Migrationsbericht der Bundesregierung (BAMF 2020) und auch in der Fachliteratur (Metzing et al. 2020) gebräuchlich ist.

## Chancen digitaler Interventionen bei Geflüchteten

Digitale Interventionen werden auch unter der Bezeichnung internet- und mobilgestützte Intervention (IMIs) zusammengefasst (Ebert und Harrer 2018). Darunter versteht man unterschiedliche Interventionsformen, die als interaktive Websites oder in Form von Apps für mobile Endgeräte gestaltet sein können (Callan et al. 2017). Einsetzen lassen sich digitale Interventionen zum einen im Rahmen einer Face-to-Face Therapie, wobei man von einer *Blended Therapy* spricht. Zum anderen können digitale Interventionen auch als *Stand Alone-*Anwendungen angewandt werden, entweder als angeleitete Interventionen, d. h. mit therapeutischer Begleitung, oder nichtangeleitet, somit ohne Therapeutenkontakt (Ebert und Harrer 2018). Digitale Interventionen können über unterschiedliche therapeutische Inhalte verfügen, wie z. B. Schreibaufgaben, eine Tagebuchfunktion oder Materialien zur Psychoedukation. Darüber hinaus umfassen viele digitale Interventionen Möglichkeiten zum Kontakt mit Therapeut*innen, wie Textnachrichten oder Videochat.

Der maßgebliche Nutzen von IMIs wird in der Ermöglichung eines niedrigschwelligen Zugangs zu Versorgungsangeboten gesehen (Ashfaq et al. 2020; Böttche et al. 2016; Burchert et al. 2019; Schneider et al. 2017; Sijbrandij et al. 2017). Es lassen sich strukturelle, kulturelle und individuelle Zugangsbarrieren unterscheiden (Böttche et al. 2016; Burchert et al. 2019; Gutknecht et al. 2020; Kupfer und Gamper 2020). Im Folgenden wird jede dieser Barrieren erläutert, und konkrete Beispiele werden angeführt, wie digitale Interventionen zu einer Überwindung dieser Barrieren beitragen können.

### Strukturelle Zugangsbarrieren

In struktureller Hinsicht ist der eingeschränkte Zugang zu medizinischen Versorgungsleistungen als Barriere für Geflüchtete zu nennen. Gesundheitsleistungen gemäß der Gesetzlichen Krankenversicherung (GKV), darunter auch psychische Versorgungsleistungen, dürfen erst nach 15 Monaten Aufenthalt in Deutschland in Anspruch genommen werden. Vor Ablauf dieser Zeitspanne sind psychische Versorgungsleistungen nur im Ausnahme- bzw. Notfall zulässig, etwa beim Vorliegen akuter Suizidgefahr (Metzing et al. 2020). Diese Regelung steht im eklatanten Widerspruch zum hohen Bedarf an psychischer Versorgung unter Geflüchteten. Eine akute Unterversorgung ist die Folge, was sich besonders an den Hochrisikogruppen zeigt. So haben nur 4 % der Jugendlichen mit Fluchterfahrung Zugang zu Versorgungsangeboten (Sukale et al. 2020). Aber auch, wenn ein Rechtsanspruch auf Versorgung besteht, beträgt die Wartezeit für Geflüchtete im Mittel sechs Monate (Sijbrandij et al. 2017). Aus klinischer Sicht sind lange Wartezeiten problematisch, da sie zu einer Verstetigung von Symptomen beitragen können (Burchert et al. 2019). Da nach biopsychosozialem Verständnis soziale und politische Determinanten die Gesundheit als Ganzes beeinflussen (Führer 2020), können strukturelle Zugangshürden nicht nur Stressoren für psychische Erkrankungen darstellen, sondern sich auf die Gesundheit insgesamt negativ auswirken. In der Überbrückung von Wartezeiten auf einen Therapieplatz haben sich digitale Interventionen bewährt (Sander et al. 2016) und ließen sich daher sinnvoll einsetzen, um die negativen Auswirkungen langer Wartezeiten für Geflüchtete zumindest abzumildern. Aus der *agency*-Perspektive würde der Einsatz digitaler Interventionen in der Wartezeit dazu beitragen, die Handlungsfähigkeit Geflüchteter zu aktivieren und sie aus der Passivität herauszuholen.

Eine weitere strukturelle Zugangsbarriere stellen die räumlichen Hürden und die oftmals eingeschränkte Mobilität von Geflüchteten dar (Schouler-Ocak 2020; Sijbrandij et al. 2017). Verschiedene digitale Interventionen können unabhängig von festen Terminen und jeweils vor Ort genutzt werden. Dadurch sind die Nutzer*innen weder zeitlich noch räumlich gebunden und können Versorgungsleistungen gemäß ihren Möglichkeiten nutzen. *Agency* ist auch hier ein zentraler Ansatzpunkt. Kompetenzen zum Selbstmanagement können durch digitale Interventionen abgerufen werden.

Ein Beispiel für das Potenzial digitaler Interventionen, strukturelle Zugangsbarrieren zu verringern, ist „Step-by-Step“ (Burchert et al. 2019; Heim und Burchert 2018). Dabei handelt es sich um eine Selbsthilfe-App, die von der Weltgesundheitsorganisation (WHO) entwickelt wurde und bei Depression eingesetzt wird. „Step-by-Step“ wird als angeleitete Selbsthilfeintervention bei Geflüchteten im Libanon eingesetzt. Die Anleitung erfolgt durch Laien mit demselben Migrationshintergrund, die in psychologischer Erster Hilfe geschult sind, sogenannte E‑Helfer (Heim und Burchert 2018). Damit erhalten Klient*innen Unterstützung durch Ansprechpartner*innen, die zwar keine ausgebildeten Therapeut*innen sind, aber technische Hilfestellung leisten können und derselben Kultur angehören. Der Einsatz der App im Libanon hat dazu geführt, mehr Personen an die therapeutische Versorgung heranzuführen (Heim und Burchert 2018). Zudem wurde die App für den Einsatz bei Geflüchteten aus Syrien adaptiert, die sich in Deutschland, Schweden und Ägypten aufhalten (Burchert et al. 2019; Heim und Burchert 2018; Sijbrandij et al. 2017).

### Kulturelle Zugangsbarrieren

Wie bereits angeführt, haben wir es hinsichtlich der Versorgungslücke nicht nur mit einem mangelnden Angebot an Versorgungsleistungen, sondern auch mit einer mangelnden Inanspruchnahme bestehender Angebote zu tun (Kupfer und Gamper 2020). In ihrer Studie kommen Sibrandij et al. (Sijbrandij et al. 2017) zu dem Ergebnis, dass 80–90 % der Geflüchteten mit Symptomen keine Versorgungsleistung in Anspruch nehmen. Ein Grund hierfür könnte in kulturell differenten Haltungen und Wahrnehmungen bestehen (Kandiah 2018). Oftmals sind Sichtweisen von psychischen Erkrankungen und Therapieformen sowie Ausdrucksweisen von Leid durch kulturelle Faktoren geprägt und weichen vom westlichen Verständnis ab (Peseschkian 2020; Preitler 2018). Konzepte wie Trauma oder Depression können unter Umständen schwierig vermittelbar sein, wenn diese in einer bestimmten Kultur unbekannt oder anders belegt sind (Kandiah 2018). Die Folge können Fehldiagnosen und nicht bedarfsgerechte Therapien sein, was zu Frustrationserlebnissen und Therapieabbruch führen kann (Bajbouj et al. 2018; Schouler-Ocak 2020). Hinsichtlich der therapeutischen Beziehung können ebenfalls kulturell differente Sichtweisen vorliegen. Die westlich geprägte Sichtweise einer auf Autonomie basierenden Beziehung kann auf Unverständnis stoßen, wenn Klient*innen aus Kulturkreisen stammen, in denen eine paternalistische oder direktive Beziehung als paradigmatisch angesehen wird (Peseschkian 2020). Auch kann eine Individualtherapie auf Ablehnung stoßen, wenn bei Klient*innen ein kulturell geprägtes Verständnis vorherrscht, nachdem Angehörige oder die Gemeinschaft in die Lösung individueller Probleme einzubeziehen sind (Kandiah 2018). Durch diese Faktoren wird auch die *agency* von Geflüchteten gehemmt, da sie ihre Ressourcen zur Selbstwirksamkeit nicht ausschöpfen können. Aus diesen Faktoren entsteht daher der Bedarf nach kultursensibler Versorgung. Die dazu notwendigen interkulturellen Kompetenzen können bei Therapeut*innen jedoch nicht immer vorausgesetzt werden (Danzinger et al. 2018; Kupfer und Gamper 2020). Dies liegt nicht zuletzt daran, dass die Vermittlung interkultureller Kompetenzen in Aus‑, Fort- und Weiterbildung noch nicht flächendeckend implementiert ist, obwohl entsprechende Leitlinien dies fordern (Haller et al. 2020; von Lersner et al. 2016). Auch wenn die Vermittlung interkultureller Kompetenzen verbessert würde, kann man von Therapeut*innen nicht erwarten, umfassende kulturelle und sprachliche Kenntnisse zu erwerben (Golsabahi-Broclawski et al. 2020). Hier könnten digitale Interventionen genutzt werden, um therapeutische Inhalte und Aufgaben in der jeweiligen Landessprache zu vermitteln und kultursensibel aufzubereiten. Dadurch könnten Geflüchtete in ihrem jeweiligen kulturellen Kontext erreicht und ihre *agency* direkt angesprochen werden.

Zudem ist die Sprachbarriere als kultureller Hindernisfaktor zu nennen. Die psychische Versorgung von Geflüchteten erfordert oftmals die Einbeziehung von Dolmetscher*innen, die vielfach nicht verfügbar sind (Bajbouj et al. 2018; Kupfer und Gamper 2020; Sijbrandij et al. 2017). Andererseits bedeutet die therapeutische Arbeit mit Dolmetscher*innen auch die Transformation der therapeutischen Dyade hin zu einer Triade (Wagner 2016). Die Einbeziehung von Dritten wird dabei von Klient*innen vielfach als störend empfunden (Sijbrandij et al. 2017). Der Einsatz von digitalen Interventionen in der jeweiligen Muttersprache könnte diese Problematik abmildern.

Ein Beispiel für eine gelungene kulturelle Adaption ist „Ilajnafsy“, ein internetbasiertes Therapieangebot für Klient*innen aus dem Irak, die an PTBS oder Depression leiden (Wagner 2016). Die Klient*innen erhalten Schreibaufgaben und verfassen Texte, die sie dann an arabischsprachige Therapeut*innen schicken. Von diesen erhalten sie individualisiertes Feedback und können mit ihnen auch über Videochat in Kontakt treten. Die kulturelle Adaption erfolgt hinsichtlich einer direktiv ausgerichteten therapeutischen Beziehung, der Bereitstellung von Inhalten in arabischer Sprache und der Einbeziehung spiritueller und religiöser Bezüge. Die Wirksamkeit von „Ilajnafsy“ hat sich in der Symptomreduktion und der hohen Zufriedenheit seitens der Klient*innen gezeigt (Wagner 2016). Allerdings muss einschränkend angemerkt werden, dass diese Intervention arabischsprachige Therapeut*innen voraussetzt, was nicht immer leistbar ist. „Ilajnafsy“ zeigt jedoch, dass es möglich ist, therapeutische Inhalte sprachlich und kulturell zu adaptieren und dabei die *agency* von Klient*innen zu aktivieren.

### Individuelle Zugangsbarrieren

Eine weitere Zugangsbarriere stellt die Angst vor Stigmatisierung dar (Kupfer und Gamper 2020; Jumaa et al. 2020; Kandiah 2018). So werden in bestimmten kulturellen Kontexten psychische Erkrankungen mit niederem sozialen Status assoziiert (Kandiah 2018). Menschen mit einer psychischen Erkrankung werden daher oft sozial ausgegrenzt. Als Folge gilt das Sprechen über psychische Symptome als gesellschaftliches Tabu. Menschen mit psychischen Erkrankungen erleben Gefühle von Scham und haben Angst davor, Schande über ihre Familie zu bringen (Zolezzi et al. 2018). Diese Faktoren halten viele Menschen davon ab, psychische Versorgungsangebote wahrzunehmen, und hemmen damit ihre *agency*. Hinzu kommt bei Geflüchteten die besondere Situation des Fremdseins und des unklaren Aufenthaltsstatus. Geflüchtete mit einer psychischen Erkrankung sind mit der Gefahr einer doppelten Stigmatisierung konfrontiert, sowohl als Geflüchtete als auch als psychisch Kranke (Jumaa et al. 2020). Da manche digitalen Interventionen anonym und oftmals auch ohne direkten Therapeutenkontakt angewendet werden können, lässt sich die Angst vor Stigmatisierung reduzieren (Böttche et al. 2016).

Ein Beispiel dafür, wie sich digitale Interventionen zur Vermittlung von *mental health literacy* sowie von Informationen hinsichtlich psychosozialer Unterstützung nutzen lassen, ist das Projekt HELP@APP, das zurzeit am Universitätsklinikum Hamburg-Eppendorf durchgeführt wird (Golchert et al. 2019). Dabei wird eine Selbsthilfe-App für Geflüchtete aus Syrien mit PTBS entwickelt, die ein Modul zur Psychoedukation enthält. In der App wird über Therapieoptionen, aber auch über den Zusammenhang zwischen Krieg, Flucht und posttraumatischem Stress aufgeklärt. Darüber hinaus enthält die App Informationen bezüglich Kontaktmöglichkeiten zu psychosozialen Hilfsdiensten.

## Risiken

Digitale Interventionen sind mit Risiken verbunden, v. a. die oftmals mangelnde Evidenzbasierung der vermittelten Inhalte (Königbauer et al. 2017), das Fehlen verbindlicher Qualitätsstandards (DGPPN 2019) und Mängel hinsichtlich Datenschutz und Datensicherheit (Huckvale et al. 2019). Ein zentraler Mangel vieler digitaler Interventionen ist die intransparente oder fehlende *privacy policy*, d. h. Regeln für den Umgang mit patientenbezogenen Daten (Huckvale et al. 2019). Dazu gehört, dass in vielen Fällen keine Aufklärung über die sekundäre Datennutzung im Sinne einer Weitergabe der Daten an Dritte erfolgt. Gemäß den Qualitätskriterien der Deutschen Gesellschaft für Psychiatrie und Psychotherapie, Psychosomatik und Nervenheilkunde (DGPPN) zum Einsatz digitaler Interventionen sollten eine gültige Datenschutzzertifizierung gemäß der Datenschutz-Grundverordnung (DSGVO) sowie eine sichere Verschlüsselung des Datentransfers Voraussetzungen der Anwendungen sein (Klein et al. 2018).

Des Weiteren besteht ein Risiko im datenorientierten (Self‑)Tracking, also der regelmäßigen Erfassung behandlungsrelevanter Daten zur Kontrolle des Behandlungserfolges (Klein et al. 2018). Um das Phänomen der *datafication*, der Reduzierung individueller Bedarfe und Narrative auf standardisierte Datenformate, zu minimieren, sollte auch bei einer *Stand Alone-*Anwendung die Möglichkeit zur Kommunikation mit Therapeut*innen bestehen.

Darüber hinaus gibt es Klientengruppen, die besonders häufig nicht von digitalen Anwendungen profitieren. Dazu gehören Personen mit bestimmten Störungsbildern bzw. gesteigerter Symptomschwere, Personen mit geringem Bildungsgrad und mangelnder technischer Affinität sowie Personen mit Migrationshintergrund (Rubeis und Ketteler 2020). Gerade bei letzterer Gruppe zeigen sich mangelnde Akzeptanz sowie hohe Abbruchraten bezüglich einer Behandlung, die eine digitale Intervention einschließt (Ince et al. 2013). Die Gründe werden in den oftmals bestehenden Sprachbarrieren, kulturell differenten Konzepten von psychischer Gesundheit und Krankheit sowie in einer kulturell begründeten Ablehnungshaltung gegenüber psychischer Behandlung gesehen (Harper Shehade et al. 2016; Ince et al. 2013; Spanhel et al. 2019). Im Folgenden werden erstens die Gestaltung der Inhalte digitaler Interventionen, zweitens deren Einsatz im Rahmen einer Behandlung hinsichtlich kulturell differenter Haltungen analysiert.

### Inhaltliche Gestaltung digitaler Interventionen für Geflüchtete

Hinsichtlich der Gestaltung von Inhalten besteht die Gefahr, kulturgebundene Haltungen und Ansichten zu missachten und damit die *agency* von Klient*innen einzuschränken. Das kann sich bereits in der sprachlichen Gestaltung ausdrücken. Die Beschreibung von Symptomen, Störungsbildern oder therapeutischen Maßnahmen basiert bei digitalen Anwendungen zumeist auf westlich geprägten Konzepten und Sprachbildern. Hier kann es zu Übersetzungsproblemen kommen, die nicht allein sprachlicher Natur sind. Manche Symptome oder Störungsbilder können auch deshalb schwer übersetzbar sein, weil sie in nichtwestlichen Kulturen schlichtweg unbekannt sind. Umgekehrt ist es oftmals schwierig, die Beschreibungen von Symptomen durch Geflüchtete in Fachterminologie zu übersetzen und gemäß den entsprechenden Manualen wie dem DSM‑5 zu kategorisieren. Man spricht hierbei von kulturgebundenen Syndromen (*culture bound syndroms*) (Kaiser und Weaver 2019). Ein bloßes Überstülpen westlich geprägter, auf dem DSM‑5 oder anderen Klassifikationssystemen basierender Kategorien kann demnach bei Geflüchteten zu Missverständnissen, Unverständnis und mangelnder Adhärenz führen. Hinsichtlich der inhaltlichen Gestaltung von digitalen Interventionen konnten Ashfaq et al. (2020) in ihrem Review zeigen, dass mangelnde kulturelle Adaption sich negativ auf die Akzeptanz und Nutzung dieser Anwendungen unter Geflüchteten auswirkt. Somit werden durch diese Mängel auch die Potenziale der *agency* von Geflüchteten nicht genutzt, ihre eigenen Beschreibungen und Erklärungsmodelle in den Behandlungsprozess einzubringen. Dabei ist auch zu beachten, dass Geflüchtete eine heterogene Gruppe darstellen. Geflüchtete aus dem subsaharischen Afrika bringen andere Konzepte und Narrative mit, als Geflüchtete aus Afghanistan oder Syrien. Digitale Interventionen müssen demnach dem jeweiligen kulturellen Hintergrund der zu behandelnden Gruppe entsprechend gestaltet bzw. eingesetzt werden.

### Einsatz digitaler Interventionen in der Behandlung

Ähnliches lässt sich hinsichtlich des Einsatzes digitaler Interventionen in der Behandlung von Geflüchteten sagen. Auch hier können, wie bereits beschrieben, kulturell differente Sichtweisen als Hemmnisfaktoren wirken. Hinzu kommt der Mangel an Therapeut*innen mit interkulturellen Kompetenzen (Kupfer und Gamper 2020). Eine wesentliche Dimension hierbei ist die kulturell differente Gewichtung von Individualismus und Kollektivismus (Peseschkian 2020). Häufig sind Klient*innen aus arabischen Herkunftsländern stärker kollektivistisch geprägt. Die Einbindung von Angehörigen in eine Behandlungssituation dient oft zur Herstellung von Vertrauen gegenüber Therapeut*innen. Eine primär auf das isolierte Individuum fokussierende Behandlung mit digitalen Interventionen kann daher zu Konflikten führen.

Ein wesentlicher Faktor in diesem Zusammenhang ist der Stellenwert der Autonomie. Das Potenzial digitaler Interventionen zum Empowerment von Klient*innen, d. h. der Stärkung selbstbestimmten Handelns und Entscheidens, wird in der Forschung häufig als positiver Aspekt hervorgehoben (Bell et al. 2017; Erbe et al. 2017; Hilty et al. 2017; Rubeis und Steger 2019). Analog zu Krankheitskonzepten ist jedoch auch das Konzept der Autonomie kulturgebunden. Ein westliches, individualistisches Verständnis von Autonomie, wie es sich auch im Prinzip des Informed Consent widerspiegelt, kann bei manchen Geflüchteten auf Ablehnung stoßen. Hierbei bietet es sich an, statt auf das individualistische Verständnis von Autonomie auf die *agency* von Klient*innen zu setzen. Das *agency*-Konzept hat den Vorteil, dass die Einbeziehung von Familie und Gemeinschaft in der Entscheidungsfindung ermöglicht wird. Die familiäre bzw. gemeinschaftliche Unterstützung lässt sich somit als Ressource begreifen und in die Behandlung integrieren.

## Perspektiven des Einsatzes digitaler Interventionen bei Geflüchteten

Digitale Interventionen haben großes Potenzial, die Versorgungssituation von Geflüchteten zu verbessern. Wie die angeführten Beispiele zeigen, wurden manche dieser Anwendungen bereits erfolgreich eingesetzt. Allerdings bestehen aus ethischer Sicht Risiken, auch hinsichtlich des *agency*-Aspekts, die es bei der Anwendung zu beachten gilt. Risiken sind die mangelnde Kultursensibilität der Anwendungen und mangelnde interkulturelle Kompetenzen von Therapeut*innen sowie der Konflikt zwischen einem individualistischen Autonomieverständnis und einem gemeinschaftlich orientierten Hilfesuchverhalten. Im Folgenden werden Perspektiven und Empfehlungen für eine *agency*-basierte therapeutische Praxis diskutiert. Ziel ist es, einen Einsatz digitaler Interventionen bei Geflüchteten zu ermöglichen, der die Risiken minimiert und das Potenzial digitaler Interventionen voll ausschöpft. Zentral ist hierbei der Ansatz einer *kultursensiblen, bedarfsorientierten* und *ressourcenorientierten* Versorgung, der die *agency* von Geflüchteten zugleich als Ressource abruft und fördert.

### Kultursensible Versorgung

Zentral für den erfolgreichen Einsatz digitaler Interventionen bei Geflüchteten sind interkulturelle Kompetenz und ein kultursensibler Ansatz (Golsabahi-Broclawski et al. 2020; Gutknecht et al. 2020; Peseschkian 2020; Schouler-Ocak 2020). Hier ist jedoch anzumerken, dass es kein verbindliches theoretisches Konzept der interkulturellen Kompetenz, nicht einmal eine einheitliche Definition gibt (Genkova 2020). Vielmehr firmieren unter dem Begriff interkulturelle Kompetenz heterogene Konzepte. Zudem erweist sich die evidenzbasierte Messung der Wirksamkeit dieser Kompetenzform als schwierig, weshalb ihre Reliabilität und Validität fraglich sind (Genkova 2020). Für die Praxis bedeutet das, einer Minimaldefinition interkultureller Kompetenz und kultursensiblen Behandelns zu folgen. Ein entsprechender Ansatz könnte darin bestehen, Klient*innen in ihrem jeweiligen kulturellen Kontext zu verstehen und dabei kulturelle Aspekte als Einflussfaktoren hinsichtlich der Therapiegestaltung und therapeutischen Beziehung anzuerkennen (Peseschkian 2020). Kulturell differente Sichtweisen stellen eine Herausforderung dar, können aber auch als Chance gesehen werden, neue Perspektiven und Handlungsmöglichkeiten zu gewinnen (Preitler 2018). Dabei ist der *agency-*Aspekt von zentraler Bedeutung. Entscheidend ist, wie Klient*innen kulturelle Normen und Sichtweisen individuell als Akteure realisieren. Das wirkt sich auch auf die Nutzung digitaler Interventionen aus. Eine Möglichkeit, der individuell gelebten kulturellen Realität zu begegnen, ist die Einbeziehung von sogenannten *idioms of distress* (Kaiser und Weaver 2019; Schouler-Ocak 2020). Darunter versteht man individuelle Beschreibungen von Symptomen und Zuständen, für welche Klient*innen kulturell geprägte Vorstellungen und Begriffe anstelle von vorgegebenen Fachbegriffen nutzen. Das hat den Vorteil, dass sich Klient*innen bei der Nutzung digitaler Interventionen, z. B. von Schreibaufgaben, im doppelten Sinne ihrer eigenen Sprache bedienen können. Therapeut*innen verlangt ein solches Vorgehen Flexibilität und Perspektivwechsel ab. Dafür kann sich eine verbesserte therapeutische Beziehung ergeben. Voraussetzung ist, dass die genutzten Interventionen die Möglichkeit bieten, *idioms of distress* zu verwenden.

### Bedarfsorientierte Versorgung: Digitale Interventionen und Stepped Care

Hinsichtlich der psychischen Versorgung von Geflüchteten wird häufig das sogenannte *Matched Care-*Modell umgesetzt (Schneider et al. 2017). Demnach hängt die Bereitstellung von Versorgungsangeboten von der Einrichtung ab, in welcher Personen sich aufhalten, und von den dort vorhandenen Ressourcen. Dieser Ansatz ist jedoch weder ressourceneffizient noch wird er den tatsächlichen Bedarfen von Personen gerecht. Als Alternative wird das gestufte Versorgungsmodell (*stepped care*) empfohlen (Böttche et al. 2016; Burchert et al. 2019; Danzinger et al. 2018; Schneider et al. 2017). Dieser Ansatz, der aus der Entwicklungshilfe stammt, zielt darauf, individuelle Versorgungsbedarfe zu ermitteln und die jeweils bedarfsgerechte Versorgungsleistung zur Verfügung zu stellen. Ziel ist das Schließen der Versorgungslücke durch möglichst ressourcenschonende und niedrigschwellige Angebote (Sukale et al. 2020). Auf der ersten Stufe steht die Deckung von Grundbedürfnissen, die Durchführung von Schutzmaßnahmen, die Bereitstellung der Basisversorgung und die Vergegenwärtigung rechtlicher Rahmenbedingungen. Die zweite Stufe zielt auf die Unterstützung durch Gemeinschaft, Familie, Jugendhilfe und Peers. Darauf bauen in der nächsten Stufe fokussierte niedrigschwellige Angebote auf. In der letzten Stufe werden spezielle Angebote bereitgestellt, z. B. eine Individualtherapie (Bajbouj et al. 2018; Sukale et al. 2020).

Auf allen Stufen dieses Modells lassen sich digitale Interventionen grundsätzlich einsetzen. Auf der ersten Stufe können sie zur Bedarfserhebung eingesetzt werden, etwa in Form mobiler Apps, die über Fragebögen zur Selbsteinschätzung verfügen. Auch ist ein präventiver Einsatz denkbar, beispielsweise in Form von psychoedukativen Apps. Idealerweise sollte diese Funktion mit Informationen über psychosoziale Dienste und andere Dienstleistungen sowie den entsprechenden Kontaktmöglichkeiten verbunden sein, wie es beim erwähnten Beispiel „HELP@APP“ der Fall ist. Gemäß dem *agency*-Konzept wird hiermit auch die Handlungsfähigkeit von Geflüchteten als Ausgangspunkt genommen und aktiviert. Auf der zweiten Stufe können digitale Interventionen genutzt werden, um die Unterstützung durch die Gemeinschaft zu aktivieren. Das erwähnte Beispiel der „Step-by-Step“-App zeigt, wie Peers als E‑Helfer geschult und in die Nutzung von Selbsthilfe-Interventionen eingebunden werden können. Somit werden nicht allein Klient*innen, sondern auch Peers unter dem Aspekt der *agency* adressiert. Auf der dritten Stufe können digitale Interventionen dazu genutzt werden, eine nichtangeleitete Intervention zur Verfügung zu stellen und damit die Wartezeit auf einen Therapieplatz zu überbücken. Auch hier wird die Passivität, die sich besonders hinsichtlich des Wartens auf einen Therapieplatz ergibt, durch die Ermöglichung von *agency* überwunden. Schließlich können digitale Interventionen auch auf der letzten Stufe im Rahmen einer gezielten, kultursensiblen Therapie eingesetzt werden, welche die individuelle kulturelle Realität von Klient*innen berücksichtigt und somit ebenfalls *agency* ermöglicht.

### Ressourcenorientierte Versorgung: Aktivierung von Ressourcen, Kompetenzen, und sozialen Netzwerken

Ein zentraler Aspekt in der Versorgung von Geflüchteten ist die Aktivierung vorhandener Ressourcen. Darunter lassen sich persönliche Ressourcen wie z. B. individuelle Coping-Mechanismen verstehen (Danzinger et al. 2018; Sukale et al. 2017). Diese Ressourcen lassen sich mithilfe von digitalen Interventionen nutzen, die auf Selbstmanagement und Selbstwirksamkeit zielen. Dadurch kann eine Behandlung auf vorhandenen Ressourcen der Handlungsfähigkeit im Sinne der *agency* aufbauen und diese für den Behandlungsprozess nutzbar machen. Als Resultat können Gefühle der Abhängigkeit und Hilfsbedürftigkeit reduziert und eine Zukunftsperspektive entwickelt werden (Danzinger et al. 2018).

Darüber hinaus können digitale Interventionen genutzt werden, um soziale Ressourcen in Form von Netzwerken und Gemeinschaften zu aktivieren. Soziale Netzwerke spielen für Menschen mit Fluchterfahrung in allen Phasen der Migration (Herkunftsland, Migrationsbewegung, Zielland) eine wichtige Rolle (Kupfer und Gamper 2020). Hinsichtlich der Postmigrationsphase lässt sich eine Korrelation zwischen posttraumatischem Stress sowie affektiven Störungen und mangelnder Integration in Freundschaftsnetzwerke nachweisen (Kupfer und Gamper 2020). Daher werden soziale Netzwerke in der Geflüchtetenhilfe häufig genutzt und sind auch ein wichtiger Faktor der psychischen Versorgung. Besonders in der Laienhilfe bzw. dem Peer-to-Peer-Modell wird die Ermöglichung eines niedrigschwelligen Zugangs zu Versorgungsangeboten gesehen (Gutknecht et al. 2020; Jumaa et al. 2020). Ein Beispiel hierfür ist das Projekt „In2Balance“ am Psychosozialen Zentrum für Flüchtlinge Düsseldorf e. V. (PSZ), bei welchem Laienhelfer*innen geschult wurden, Psychoedukation und Ressourcenaktivierung zu leisten und *Red Flags* für Behandlungsnotwendigkeit zu erkennen (Gutknecht et al. 2020). Die Laienhelfer*innen konnten zur kultursensiblen Wissensvermittlung und zur Überwindung von Sprachbarrieren beitragen. Befragungen in der Einrichtung ergaben, dass mehr als die Hälfte der Geflüchteten negatives Stresserleben sowie Symptome für Angst und Depression zeigten (Gutknecht et al. 2020). Die Laienhelfer*innen konnten diese Fälle erkennen und der Versorgung zuführen. Sowohl Patient*innen als auch Mitarbeiter*innen in der Einrichtung bewerteten die Maßnahme als positiv. Ein vergleichbares Projekt an der Charité hat ebenfalls positive Outcomes hinsichtlich Stärkung der Selbstwirksamkeit, Verbesserung der Bewältigungsstrategien und Symptomreduktion gezeigt (Jumaa et al. 2020). Die Erfahrung mit diesen Projekten zeigt, dass zentrale Faktoren für die Umsetzung des Peer-to-Peer-Modells engmaschige Betreuung, Koordination, Supervision und die kurzfristige Qualifizierung von Peers sind (Gutknecht et al. 2020). Da die entsprechenden Ressourcen nicht immer zur Verfügung stehen, könnten digitale Interventionen genutzt werden, die oftmals ein einfaches und ressourcensparendes Monitoring erlauben. Zudem lassen diese sich, wie das Beispiel „Step-by-Step“ zeigt, gut in die Peer-to-Peer-Betreuung integrieren.

Eine Möglichkeit hierzu wäre, digitale Interventionen im Rahmen eines *Stepped-and-Collaborative-Care-Modells *(SCCM) einzusetzen. Dabei wird das beschriebene gestufte Versorgungsmodell stärker auf die Integration von sozialen Netzwerken, Peers und Laienhelfer*innen ausgerichtet (Schneider et al. 2017). Das SCCM kombiniert demnach *stepped care* mit Peer-to-Peer-Modellen. Dies ermöglicht eine bessere Nutzung vorhandener Kompetenzen zur gemeinsamen Entwicklung von Maßnahmen (Danzinger et al. 2018). Zudem entstehen durch die Zusammenarbeit von Geflüchteten Gefühle der Gemeinschaft und Zugehörigkeit, die zu einer Destigmatisierung beitragen können (Sukale et al. 2020). Digitale Interventionen könnten genutzt werden, um durch Informations- und Kommunikationsfunktionen den Austausch von Informationen sowie die Vernetzung zu ermöglichen. Des Weiteren können Laienhelfer ihre sprachlichen und kulturellen Kompetenzen einbringen, um den Einsatz digitaler Interventionen zu begleiten. Wünschenswert wäre es, wenn diese Kompetenzen auch für die kultursensible Gestaltung von digitalen Interventionen genutzt würden.

## Conclusio

Aus ethischer Sicht haben digitale Interventionen großes Potenzial, die Versorgungssituation von Geflüchteten zu verbessern. Die Analyse der Chancen und Risiken hat jedoch gezeigt, dass sowohl die Gestaltung der Inhalte als auch der therapeutische Einsatz verbesserungsbedürftig sind. Hier bedarf es einerseits weiterer empirischer Daten, andererseits der konzeptuellen Forschung, um digitale Interventionen kultursensibel gestalten zu können. Zudem müssen interkulturelle Kompetenzen stärker in der Aus‑, Fort-, und Weiterbildung verankert werden. Unter dem Aspekt der *agency* ergibt sich für digitale Interventionen in der Versorgung von Geflüchteten eine besonders vielversprechende Perspektive. Diese Interventionen können dazu beitragen, die passive Opferrolle zu überwinden und die Handlungsfähigkeit von Geflüchteten zu stärken. Dafür bietet sich besonders eine bedarfs- und ressourcenorientierte Herangehensweise im Sinne des SCCM an, die vorhandene persönliche Ressourcen aktiviert und digitale Interventionen in bestehende soziale Netzwerke integriert. Im Rahmen des vorliegenden Beitrags konnte in dieser Hinsicht nur eine prospektive und konzeptuelle Analyse geleistet werden. Inwieweit sich die hier vorgebrachten Empfehlungen in der therapeutischen Realität umsetzen lassen, müssen empirische Studien zeigen. Daher ist vorliegender Beitrag vorrangig als Orientierungshilfe und Handlungsrahmen zu verstehen.

Auch wenn im Einsatz digitaler Interventionen ein Potenzial für die Verbesserung der psychischen Versorgung gesehen werden kann, ist jedoch ein *caveat* geboten. Es wäre ein falscher Ansatz, würde man in digitalen Technologien die Lösung sozialer, politischer oder rechtlicher Probleme sehen. Die Versorgungssituation von Geflüchteten stellt einen derartigen Problemkomplex dar. Der Einsatz von Technologie allein kann hier keine Lösung herbeiführen, sondern es bedarf dazu politischer und zivilgesellschaftlicher Bemühungen. Allerdings können digitale Technologien dazu verwendet werden, bestimmte Versorgungsleistungen zu verbessern und den Zugang zu diesen Versorgungsleistungen zu erleichtern. Somit können sie einen Beitrag zur Verbesserung der psychischen Versorgung von Geflüchteten leisten. Eine allgemeine Verbesserung der Versorgungssituation ist allerdings eine gesellschaftspolitische Aufgabe. Digitale Technologien können hierfür zwar ein wichtiges Hilfsmittel darstellen, sind jedoch keine Alternative zu notwendigen strukturellen Veränderungen im Versorgungssystem.
